# Association of Patterns of Mild Traumatic Brain Injury with Neurologic Deterioration: Experience at a Level I Trauma Center

**DOI:** 10.7759/cureus.5677

**Published:** 2019-09-17

**Authors:** Tapan Kavi, Ahmed Abdelhady, James DeChiara, Emily Lubas, Khodeja Abdelhady, Rrita Daci, Janika San Roman, Urvish K Patel

**Affiliations:** 1 Neurology, Cooper Neurological Institute, Cooper University Hospital, Camden, USA; 2 Neurology, Cooper Medical School of Rowan University, Camden, USA; 3 Internal Medicine, Washington University of Health and Science, San Pedro, BLZ; 4 Neurosurgery, University of Massachusetts, Worcester, USA; 5 Trauma, Cooper University Hospital, Camden, USA; 6 Neurology and Public Health, Icahn School of Medicine at Mount Sinai, New York, USA

**Keywords:** mild traumatic brain injury, neurologic deterioration, glasgow coma score, subdural hematoma, contusion, subarachnoid hemorrhage

## Abstract

Introduction: There are about 2.5 million emergency room visits for traumatic brain injury (TBI) every year and 75%-95% of all TBI patients have mild TBI. Previous studies have suggested that a large proportion of mild TBI patients can be treated in a non-aggressive manner, but they have not differentiated mild TBI as per radiological patterns to help in the selection of these patients. Our study aimed to identify different patterns of mild TBI to determine if certain injuries make patients more prone to neurologic worsening than others, and thus require more intensive monitoring. We also studied the factors associated with neurologic deterioration.

Methods: We conducted a retrospective study using an institutional trauma database to identify TBI patients between the years of 2015 and 2016 with admission Glasgow Coma Score (GCS) of 13 to 15, through chart review by the investigators. Radiological and neurological worsening was determined through computed tomography (CT) scan results, GCS scores, and the requirement for neurosurgical intervention. We identified the prevalence of demographic characteristics, radiological patterns, and risk factors. We studied neurologic deterioration (decline in GCS to less than 13 at 48 hours or earlier after admission) and surgical intervention among patients with different radiological patterns of TBI. We further studied the cohort of isolated subdural hematoma (SDH) patients requiring surgery to evaluate the associated risk factors.

Results: Out of 374 patients with mild TBI (mean age was 63 years), 59% were male, 77% were Caucasian, the median GCS was 15, majority of patients had isolated SDH (45%), and mixed pattern of hemorrhage (39%); the use of antiplatelet (33%) was the most commonly identified risk factors. Overall 7% of patients were found to have neurologic deterioration (GCS to less than 13) and 9% required surgical intervention at 48 hours or earlier after admission. The most common pattern of TBI requiring surgical intervention was isolated SDH (85%). Among the cohort of patients with isolated SDH, 17% required surgical intervention and 69% of those isolated SDH patients requiring surgery had neurologic deterioration. The most common risk factor in isolated SDH patients requiring surgery was antiplatelet use (34%), anticoagulant use (20%), alcohol abuse (17%), severe renal failure (17%), and thrombocytopenia (7%). Mean size of SDH in patients requiring surgery was 1.6 cm with 0.8 cm of midline shift.

Conclusion: This study identified the pattern of mild TBI associated with neurological worsening at our Level I Trauma Center. Among patients with mild TBI, SDH patients seem to be at highest risk for deterioration and requirement for surgery. If these results can be externally validated through a multi-center study, these patients could be selectively identified for aggressive monitoring in the intensive care unit (ICU) and repeat CT scans.

## Introduction

As per the Centers for Disease Control and Prevention (CDC), there are about 2.5 million emergency room (ER) visits for traumatic brain injury (TBI) every year and lifetime economic cost of TBI was estimated to be around 76 billion US dollars [1-2]. Attending to TBI patients results not just in tremendous financial burden but also heavy utilization of time and resources. 75%-95% of all TBI patients have mild TBI Glasgow Coma Score (GCS) of 13-15] [3]. Multiple studies have shown that mild TBI does not lead to neurologic deterioration and that intensive care unit (ICU) admission and repeat computerized tomography (CT) scans may not be needed in these patients [4-8]. Very few studies, however, differentiate mild TBI into radiological patterns of subdural hematoma (SDH), contusion, subarachnoid hemorrhage (SAH), and other patterns to identify the ones associated with neurologic worsening [4,8-9]. Such studies are important for significant cost reduction of mild TBI management by the targeted use of resources for patterns associated with worsening.

We studied these different patterns of mild TBI to determine if certain injuries are more prone to neurologic worsening than others, and thus require more intensive monitoring. We also studied the factors associated with neurologic deterioration.

## Materials and methods

This study was conducted at Cooper University Hospital, a Level 1 trauma center, Camden, NJ. We used the institutional trauma database to identify TBI patients (≥ 18 years old) between the year 2015 and 2016 with admission GCS of 13 to 15. Our database categorizes TBI patients into mild, moderate or severe using admission GCS scores. Patients were confirmed to be GCS 13-15 on presentation through chart review by the investigators. Radiological and neurological worsening was determined through computed tomography (CT) scan results, GCS scores, and by the requirement for neurosurgical intervention. We also collected data on antiplatelet use, anticoagulant use, alcohol use, renal function, and platelet count as possible risk factors for worsening TBI. All data collection was verified for accuracy by investigator TK. We identified the prevalence of demographic characteristics, radiological patterns (isolated SDH, isolated contusion, isolated SAH, isolated epidural hematoma (EDH), and mixed pattern), and risk factors (antiplatelet use, anticoagulation use, alcohol abuse, stage 4 and 5 chronic kidney disease or hemodialysis dependent, and thrombocytopenia-platelet count < 100,000 per microliter).

The neurologic deterioration was defined for this study as a decline in GCS to less than 13, which is a moderate TBI category at 48 hours or earlier after admission. Amongst such patients, we identified the prevalence of patients with neurologic deterioration and surgical intervention. Among patients who needed surgical intervention, we evaluated radiological patterns requiring surgical intervention. We further studied the cohort of isolated SDH patients requiring surgery to evaluate the associated risk factors.

## Results

A total of 400 patients were included from the institutional database; 26 patients had to be excluded as they were miscategorized as mild TBI. As seen in Table 1, the mean age for mild TBI patients was 63 years with 59% male, and 77% Caucasian. Median GCS score in the study was 15, suggestive of very mild pattern of TBI. Isolated SDH (45%) and mixed pattern of hemorrhage (39%) formed the majority of patients in the study. Among the risk factors, antiplatelet use (33%) was most common followed by alcohol abuse, anticoagulant use, severe renal failure, and thrombocytopenia (Table 2).

**Table 1:** Demographics and baseline features for mild traumatic brain injury patients Mild traumatic brain injury was defined as GCS 13-15 on presentation. TBI: traumatic brain injury; GCS: Glasgow Coma Score; SDH: subdural hematoma; SAH: subarachnoid hemorrhage; EDH: epidural hemorrhage.

Number of patients	374
Age (Mean)	63 years (18-99 years)
Male	59%
Caucasian	77%
African American	13%
Hispanic	8%
Initial GCS (Median)	15
Penetrating TBI	1.6%
Isolated SDH	45%
Isolated SAH	6%
Isolated Contusion	7%
Isolated EDH	3%
Mixed pattern TBI	39%

**Table 2:** Risk factors in mild traumatic brain injury (TBI) patients

Number of patients	374
Antiplatelet use	33%
Alcohol abuse	20%
Anticoagulation use	10%
Stage 4, 5 Chronic Kidney Disease or Hemodialysis dependent	7%
Thrombocytopenia: Platelet count < 100,000 per microliter	4%

Table 3 describes neurologic deterioration and neurosurgery utilization as per the pattern of mild TBI. We defined neurologic deterioration for this study as a decline in GCS to less than 13, which is moderate TBI category at 48 hours or earlier after admission. Overall 7% of patients were found to have neurologic deterioration and 9% required surgical intervention. By far, the most common pattern of TBI requiring surgical intervention was isolated SDH (85%). Depressed skull fractures, epidural hemorrhage, and mixed pattern with predominant SDH formed the remaining minority of the patients requiring surgical intervention. Of all isolated SDH patients with mild TBI, 17% required surgical intervention and none with isolated SAH or contusions required surgery. 18% of isolated EDH patients and 1% of mixed pattern of TBI required surgery.

**Table 3:** Neurologic deterioration and neurosurgery as per the pattern of mild traumatic brain injury * Neurologic deterioration was defined by a decline in GCS to less than 13 at 48 hours or earlier after admission. @ Other patterns of TBI: depressed skull fractures, epidural hemorrhage, mixed pattern of TBI, and predominant SDH. TBI: traumatic brain injury; GCS: Glasgow Coma Score; SDH: subdural hematoma; SAH: subarachnoid hemorrhage; EDH: epidural hemorrhage.

Number of patients	374
Neurologic deterioration	7%*
Surgery intervention requirement	9%
Surgery patients with isolated SDH	85%
Surgery patients with other patterns of TBI	15%^@^
isolated SDH patients required surgery	17%
isolated SAH patients required surgery	0%
isolated contusion patients required surgery	0%
isolated EDH patients required surgery	18%
Mixed pattern patients required surgery	1%

Table 4 mentions the risk factors associated with isolated SDH patients requiring surgery. Neurologic deterioration was seen in 69% of all isolated SDH patients requiring surgery. The most common risk factor in isolated SDH patients requiring surgery was antiplatelet use (34%) which was also the most common risk factor present in all mild TBI patients in this study (33%). This was followed by anticoagulant use (20%), alcohol abuse (17%), severe renal failure (17%), and thrombocytopenia (7%). Mean size of SDH in patients requiring surgery was 1.6 cm with 0.8 cm of midline shift.

**Table 4:** Risk factors in isolated subdural hematoma patients requiring surgery iSDH: isolated subdural hematoma.

iSDH requiring surgery	17%
iSDH patients requiring surgery seen to have neurologic deterioration	69%
iSDH requiring surgery on antiplatelets	34%
iSDH requiring surgery on anticoagulants	20%
iSDH requiring surgery found to have thrombocytopenia of < 100K	7%
iSDH requiring surgery with alcohol abuse	17%
iSDH requiring surgery with stage 4, 5 Chronic Kidney Disease or Hemodialysis dependent	17%
Mean size of SDH in those requiring surgery	1.6 cm largest transverse diameter and 0.8 cm midline shift

## Discussion

There have been previous studies looking at mild TBI and patterns that may not need ICU admission. Washington et al. and Alahmadi at al. showed that mild TBI patients with a convexity SAH, small convexity contusion, small intraparenchymal hemorrhage (= 10 ml), and/or small SDH have low rates of hemorrhagic progression and may not need ICU admission [4] and patients with an initial GCS score of 15 or an initial contusion size < 14 ml may not require delayed evacuation [10]. Other studies have shown that routine repeat head CTs may not be needed in patients with a minimal head injury as clinical deterioration often precedes deterioration on CT scans [11]. GCS score lower than 15, multiple traumatic brain lesions on first CT, age over 65 years, and an interval of fewer than 90 minutes between arrival to the hospital and first head CT were identified as risk factors for worsening in mild TBI patients [11]. Very early head CTs may not capture the actual extent of TBI as was also shown by Oertel et al. They have shown that nearly half of TBI patients with CT within two hours of injury have early progressive hemorrhage [12].

The major result from the study is that isolated SDH is the most likely pattern of TBI to be associated with neurologic deterioration or need for neurosurgical intervention. In our study, the only other patterns of TBI requiring surgery were EDH, depressed skull fractures, and mixed pattern with predominant SDH. 17% of isolated SDH patients with mild TBI required surgery, and 69% of these operated patients had neurologic deterioration. The fact that not all operated patients had neurologic deterioration suggests that it can be an institutional practice to operate on patients with mild TBI based on the size of SDH. The mean size of SDH in these operated patients was 1.6 cm. Previous studies showed that those with SDH less than 10 cm^3^ were not seen to have neurologic deterioration or requirement for surgery and hence do not need for ICU monitoring [4,13]. Patients with less than 10 cm^3^ of SDH but with concomitant ICH or multiple ICHs had neurologic deterioration as well though much lower rate than those with SDH more than 10 cm^3^ [14]. It is unclear as to why SDH is associated more than neurologic deterioration and requirement for surgery as compared to other patterns of TBI.

Isolated SAH patients in our study were seen to be very benign and none of these patients required surgery. This is in agreement with previous studies [15-16]. Nassiri et al. found through meta-analysis that although 5.7% of patients had the progression of lesions on interval imaging, only 0.75% experienced neurological deterioration and 0.0017% eventually required specialized neurosurgical services [16]. Gates et al., in fact, found that radiographic progression and clinical deterioration of isolated SAH was low despite anticoagulation and/or antiplatelet medication use [17].

Isolated contusions in our study were also not associated with neurologic deterioration or requirement for surgery. Previous studies have found that older age, anticoagulant use, antiplatelet medication use, and frontal contusion location have been associated with hemorrhagic progression of a contusion [18]. Allison et al. derived a score to predict hemorrhagic progression of contusion and found that subdural hemorrhage, SAH, and skull fracture were predictive of progression [19]. Size of contusion also seems to be associated with progression, but these patients were not strictly of the mild degree for TBI. In our study, all mild TBI with isolated contusions were benign and did not require surgery even in the presence of risk factors.

The study does have limitations as any retrospective study, as some data could have been missing or misinterpreted. The fact that not all SDH patients in this study had neurologic deterioration before surgery could represent a practice pattern specific to our institute as well wherein a more aggressive approach is used for treating SDH patients as compared to other patterns. Also, radiographically it is often difficult to ascertain with certainty that isolated SDH, SAH or contusions are present as the co-occurrence of different patterns of TBI (multi-compartment) is very common. For example, it is often very difficult to exclude a very small amount of SAH in patients with predominant SDH or contusions. Nevertheless, this study does have a large number of patients at a single institute and is one of the first ones to study individual radiological patterns of TBI and their association with neurologic worsening.

## Conclusions

This study presents experience with management of mild TBI at a high volume Level I Trauma Center. Among different radiological patterns of mild TBI, the need for surgical treatment or neurological worsening was seen predominantly in SDH patients in this large-sized single-center study. Patients with mild TBI and isolated SAH or contusions did not have neurologic deterioration in accordance with previous studies. If the results of this study can be externally validated, recommendations can be made for selective close monitoring of mild TBI patients with SDH pattern only.
